# Association between Timing of Energy Intake and Insulin Sensitivity: A Cross-Sectional Study

**DOI:** 10.3390/nu12020503

**Published:** 2020-02-16

**Authors:** Vittobai Rashika Rangaraj, Alankrita Siddula, Helen J. Burgess, Silvana Pannain, Kristen L. Knutson

**Affiliations:** 1 Department of Medicine, The University of Chicago, Chicago, IL 60637, USA; virangaraj@luriechildrens.org (V.R.R.); spannain@medicine.bsd.uchicago.edu (S.P.); 2 Center for Circadian and Sleep Medicine, Department of Neurology, Northwestern University Feinberg School of Medicine, Chicago, IL 60611, USA; asiddula1@gmail.com; 3 Sleep and Circadian Research Laboratory, Department of Psychiatry, University of Michigan, Ann Arbor, MI 48109, USA; bhelen@med.umich.edu

**Keywords:** circadian rhythm, insulin resistance, diet habits, Type 2 Diabetes

## Abstract

In addition to the caloric and macronutrient composition of meals, timing of energy consumption may be important for optimal glucose metabolism. Our goal was to examine whether the habitual timing of energy intake was associated with insulin sensitivity in healthy volunteers. Volunteers without diabetes aged 21–50 years completed a 3-day food diary and underwent an oral glucose tolerance test to estimate insulin sensitivity (*n* = 44). From the food diary, we calculated the proportions of the total energy and macronutrients consumed in the morning and evening, and the clock time at which 25%, 50% and 75% of total energy was consumed. A greater proportion of energy intake in the morning was significantly associated with higher insulin sensitivity estimated by Matsuda Index (B = 2.8 per 10%; 95%CI: 0.3, 5.2). The time at which 25% of energy was consumed was associated with insulin sensitivity estimated by Matsuda Index (B = −1.6 per hour; 95%CI: −3.0, −0.3) and QUICKI (B = −1.4 per hour, 95%CI: −2.8, −0.1). The timing of carbohydrate consumption demonstrated similar associations. Greater energy intake earlier in the day was associated with higher insulin sensitivity in individuals without diabetes.

## 1. Introduction

Insulin resistance is a central aspect in the etiology of type 2 diabetes and a strong predictor of future development of diabetes [1]. Major causes of insulin resistance include excess weight and physical inactivity [2,3], however, identifying novel predictors of insulin resistance may help better understand diabetes etiology and ultimately reduce risk of the disease. One such novel predictor may be the timing of food intake. Although total energy intake and macronutrient composition are associated with metabolic health [4,5,6], when someone eats may also be important. The timing and distribution of energy intake across the day may be important because feeding is a major synchronizer of the biological (circadian) clocks in peripheral organs and tissues [7]. Therefore, inappropriate timing of feeding may disrupt circadian physiology and lead to impaired glucose metabolism.

Circadian disruption induced in controlled laboratory studies in healthy volunteers resulted in impaired glucose tolerance and increased insulin resistance [8,9]. In these experiments, feeding at the “wrong” time was likely a key-contributing factor because the feeding occurred at a time when the central circadian clock did not anticipate food intake. Therefore, energy intake at an inappropriate circadian time could lead to adverse metabolic dysfunction. Shift workers, i.e., individuals who are often awake at night and sleeping during the day, experience extreme circadian disruption and adverse meal timing, and shift workers have increased risk of metabolic dysfunction [10,11,12,13]. The majority of adults, however, are not shift workers—and therefore it is important to understand the degree to which meal timing is associated with metabolic function outside of extreme circadian disruption, such as shift work. One study of young college-aged adults found that later timing of energy intake was associated with greater body fat [14] and greater body fat is a risk factor for insulin resistance and diabetes. The aim of this study was to test the hypothesis that the later timing of energy intake will be associated with higher estimated insulin resistance in a group of individuals without any form of diabetes and who are not shift workers.

## 2. Methods

### 2.1. Study Design

Volunteers between the ages of 21 and 50 years were recruited to participate in a study about sleep, circadian rhythms and diabetes risk through flyers and online advertising. In this analysis, 52 volunteers were recruited and completed the study between April 2013 and November 2016 at the University of Chicago. Inclusion criteria were African-Americans and whites, non-smokers and the absence of any major illness including diabetes, morbid obesity (body mass index ≥ 40 kg/m^2^), cardiovascular disease and sleep disorders. Exclusion criteria included anyone who tested positive for the abuse of any common drugs, worked night shifts or were taking medications other than antihypertensive or lipid lowering medication. Participants who travelled across time zones were studied only after they remained in the Central Time Zone for one month prior to the study. All participants gave written informed consent prior to participation. The study was conducted in accordance with the Declaration of Helsinki, and the protocol was approved by the institutional review board at the University of Chicago, Chicago, Illinois.

The participants were screened during an overnight laboratory stay, which included full polysomnography to rule out moderate or severe sleep disordered breathing (apnea hypopnea index ≥ 15 events/h) or other sleep disorders and a 2-h oral glucose tolerance test (2 h OGTT) to rule out diabetes. Individuals who met criteria for prediabetes at the 2 h-OGTT were not excluded. Eligible participants were then asked to continuously wear a waterproof wrist actigraphy monitor for 10 days and complete a 3-day food diary on days 7–9. Within one week of the completion of the 10-day at-home session, the participants were admitted to the University of Chicago sleep research laboratory and Clinical Research Center in the early evening (between 17:00 and 18:30) and began fasting at 20:00 when only water was allowed. The participants were given a 10-h bedtime opportunity from 22:00 to 8:00. A 5-h 12-sample oral glucose tolerance test (5 h OGTT) was performed beginning at 9:00 the following morning. Their age and gender were recorded, and height and weight were measured. Body mass index (BMI) was calculated as kg/m^2^. All procedures were identical in all subjects. Further, researchers were blind to subject characteristics when performing the assessments of the key outcomes, including the glucose and insulin assay as well as estimation of insulin sensitivity, and the calculations of the exposure variables, i.e., meal timing.

### 2.2. Insulin Sensitivity Assessment

The 5 h OGTT began in the morning after a 12-h overnight fast. An intravenous catheter was placed in the distal forearm and two baseline blood samples were collected (−10 and 0 min) after which 75 g dextrose dissolved in 296 mL of orange-flavored water (Trutol 75, Thermo Scientific, Middletown, VA, USA) was ingested orally within 5 min by the participant. Additional blood samples were collected at 10, 20, 30, 60, 90, 120, 150, 180, 240 and 300 min post-ingestion [15]. The 5-h test with fasting plus 10 samples post-glucose ingestion was chosen because a previous validation study indicated these samples were as accurate as a test with 22 samples [15]. Plasma glucose was measured using a 2300 STAT PLUS analyzer (Yellow Springs Instruments, Yellow Springs, OH, USA), while serum insulin was measured using IMMULITE 2000 (Siemens, Malvern, PA, USA) on all 12 blood samples. 

In this study, we used two OGTT-derived insulin sensitivity indices; Matsuda Index and QUICKI. Matsuda Index represents both hepatic and peripheral insulin sensitivity as it includes the dynamic changes in fasting and postprandial glucose and insulin levels, while QUICKI represents hepatic insulin sensitivity since it uses only fasting insulin and glucose measures [16]. Fasting glucose and insulin concentrations were calculated as an average of the −10 and 0-min readings. Insulin sensitivity by Matsuda Index was calculated as 10,000/sqrt [(fasting glucose (mg/dL) × fasting insulin (μU/mL)) × (mean glucose × mean insulin during the OGTT)] and QUICKI was calculated as [1/(log (fasting glucose) (mg/dL) + log (fasting insulin) (μU/mL))] [17,18,19,20]. We calculated two Matsuda Index values, one using 10 data points until the 180-min time point and the other using 12 data points until the 300-min time point. Many prior studies that used OGTT to calculate the Matsuda Index had only 120 or 180 min of sampling, and therefore we wanted to have values that were comparable to previous studies. Furthermore, two participants had glucose values that dropped below 50 mg/dL after the 180-min sample and the test was terminated to avoid hypoglycemia, while 2 other participants terminated the glucose test voluntarily before the completion of the 300-min blood sample. Thus, 4 participants have a Matsuda Index calculated from 180 min rather than 300 min of sampling.

### 2.3. Assessment of Meal Timing

Participants completed a self-administered prospective diary of food and drinks consumed on 3 days, which included a combination of weekdays and weekend days to determine habitual energy intake [21]. A prospective method is generally more accurate because it does not rely on participant recall [22]. In addition, more than 3–4 days of diary recording has been demonstrated to be too burdensome and data quality declines with more days [22]. The food diary required participants to record the description of the food/drink, the amount of food consumed, and the clock time consumption began. Participants classified each meal as either breakfast, lunch, dinner or snack. To provide clarity and ensure consistency, the food diary included detailed instructions about the type of information the participant is required to record and these instructions were explained in-person prior to the start of the food recording period and completed food logs were later reviewed by a trained study coordinator. The food log information was entered into the Food Processor Nutrition Analysis Software ESHA (version 10.7.0, Salem, OR) to calculate calories and macronutrient content (grams of carbohydrates, protein and fat). We calculated the total amount of energy intake (kilocalories, Kcal) and macronutrients for each day. Each meal was classified as an individual eating event if the energy intake was greater than 5 Kcal and the time between two meals was at least 15 min.

We calculated two measures to represent the timing of energy intake consumed across the day. First, the proportions of total energy intake and macronutrient consumption were estimated for three periods in each day based on participant-classified meal types: morning (breakfast + morning snacks), afternoon (lunch + afternoon snacks) and evening (dinner + evening snacks). Second, because the identification of meal type is subjective and independent of time of day, we calculated the clock time at which the participant reached 25%, 50% and 75% of their total daily energy intake. We chose these cut offs because they are similar to commonly-used percentiles, such as those used in boxplots of data. These calculations were conducted for each food diary day and then averaged across all days. 

### 2.4. Covariates

In our analyses, we adjust for the following possible confounders: age, sex, body mass index (BMI), race, total daily energy intake and sleep timing. Age, sex and race were self-reported. BMI was calculated based on height and weight, which was measured by trained research staff. Total energy intake was calculated using the food diary as described above. Finally, sleep timing was calculated based on 7–10 days of wrist activity monitoring. Participants wore waterproof wrist actigraphy monitors (Actiwatch-2 or Actiwatch Spectrum, Respironics/Philips, Bend, OR) on their non-dominant wrist continuously. The subjects were asked to press an event marker button on the monitor when they went to bed and when they awoke, and they also completed a short sleep log each morning. Both the event marker and log were used to identify the bed time and wake time for the analysis of sleep periods. We used the associated Actiware software (v. 6.0) to calculate average sleep onset, sleep end and the midpoint between these two time points. Three participants were missing valid actigraphy data due to technical problems in the device or recorded data.

### 2.5. Statistical Analysis 

Among the 52 participants who completed the study, 4 had incomplete food diary data, 3 were missing actigraphy data, and 1 had invalid OGTT measures and were excluded, leaving 44 subjects in the primary analysis. Means and standard deviations of all continuous variables and proportions of categorical variables were calculated. We also examined whether the distribution of each variable approximated a normal distribution. We identified two variables that had highly skewed distributions: the time 75% of total calories was consumed and the time 75% of total fat was consumed. Analyses of these variables were repeated using the natural log transformation. The insulin sensitivity and dietary timing measures were treated as continuous variables. Unadjusted associations between the dietary timing measures and the measures of insulin sensitivity were estimated using simple linear regression. Multiple linear regression models were used to estimate the associations between insulin sensitivity and the measures of dietary distribution and timing variables adjusting for age, sex, BMI, race, total daily energy intake, and the midpoint of sleep period as a measure of sleep timing. We also ran models using the Matsuda Index calculated in those with complete data for all 300 min. Finally, since insulin sensitivity exhibits diurnal variations that decrease during sleep [23], we performed sensitivity analyses that excluded those whose habitual wake time based on the week of actigraphy was later than the time the OGTT started (*n* = 5). Statistical analysis was performed using STATA (version 14.2).

## 3. Results 

In this sample, average age was approximately 30 ± 8 years, approximately half (48%) of the sample identified as female and 39% of the sample was African American. A description of the demographic, glucose and sleep characteristics of the study participants is shown in Table 1. Table 2 summarizes the dietary variables. The participants consumed 2411 ± 744 Kcal each day on average (Table 2). Only 17% (*n* = 8) of the participants had three or fewer eating events each day, while the remaining participants had four to five eating events each day on average. When the eating events (main meals and snacks) were grouped into three categories—morning, afternoon and evening—the average proportion of energy intake in the morning was 23% ± 12%, whereas the average proportion of energy intake in the evening was 41% ± 13%. The proportions of macronutrient consumption had similar patterns with greater proportions consumed in the evening (Table 2). The average clock times at which participants reach 25% of their total energy intake was approximately noon (±2 h) on average and 75% of total energy intake was reached by approximately 18:30 (±1.5 h) on average (Table 2). Similar times were observed for macronutrient consumption.

### 3.1. Proportions of Energy Intake in Morning Versus Evening

**Figure 1:**
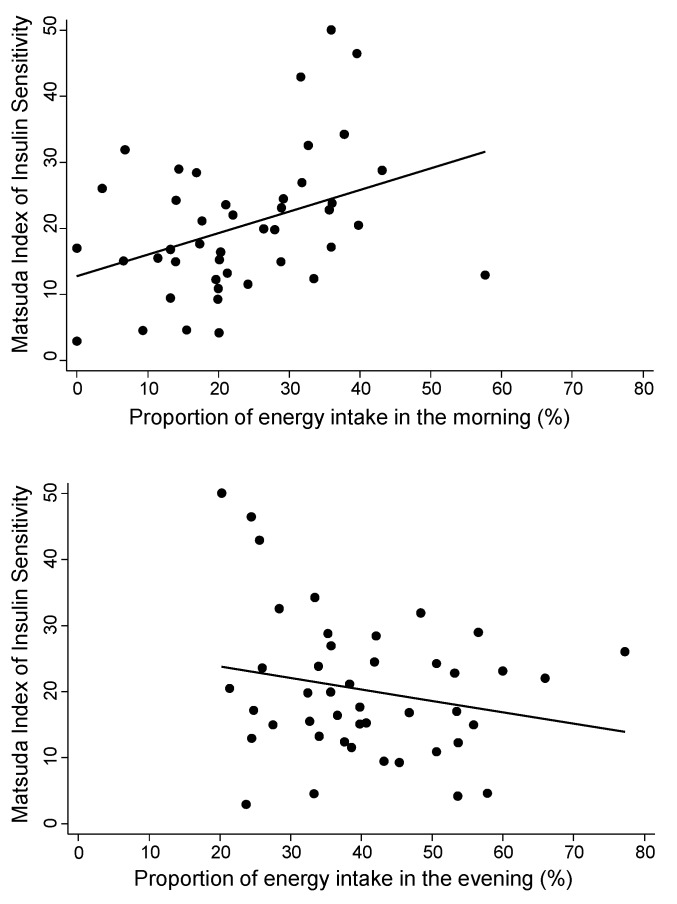
Associations between the Matsuda Index of insulin sensitivity and the proportion of energy intake in the morning (**top panel**) and the proportion of energy intake in the evening (**bottom panel**). The unadjusted regression line is plotted. The r values represent unadjusted Pearson correlations and associated *p* values.

Consuming a greater proportion of energy in the morning was significantly associated with higher insulin sensitivity estimated by both Matsuda Index and QUICKI (Table 3). Figure 1 illustrates the unadjusted associations between insulin sensitivity based on Matsuda Index and the proportion of energy intake consumed in the morning (top panel) and evening (bottom panel). The results from the fully adjusted regression model indicate that 10% greater energy intake in the morning would be associated with a 2.8-unit-higher Matsuda Index on average. The proportion of energy intake consumed in the evening was negatively associated with insulin sensitivity but was not statistically significant. Using Matsuda Index at 300 minutes (*n* = 40) produced similar results (see Supplementary Table S1). 

When examining the three macronutrients, carbohydrates, protein and fat, a greater proportion of carbohydrates consumed in the morning was significantly associated with higher insulin sensitivity based on the Matsuda Index (B = 2.9, 95%CI 0.8, 5.1, *p* = 0.008 in adjusted model) and based on QUICKI (B = 0.006, 95%CI 0.001,0.012, *p* =0.02 in adjusted model). The proportion of fat consumed in the evening was negatively associated with insulin sensitivity based on the Matsuda Index (B = −2.1, 95%CI −4.0, −0.2, *p* = 0.029 in adjusted model). 

### 3.2. Clock Times at Which Proportions Are Reached

**Figure 2:**
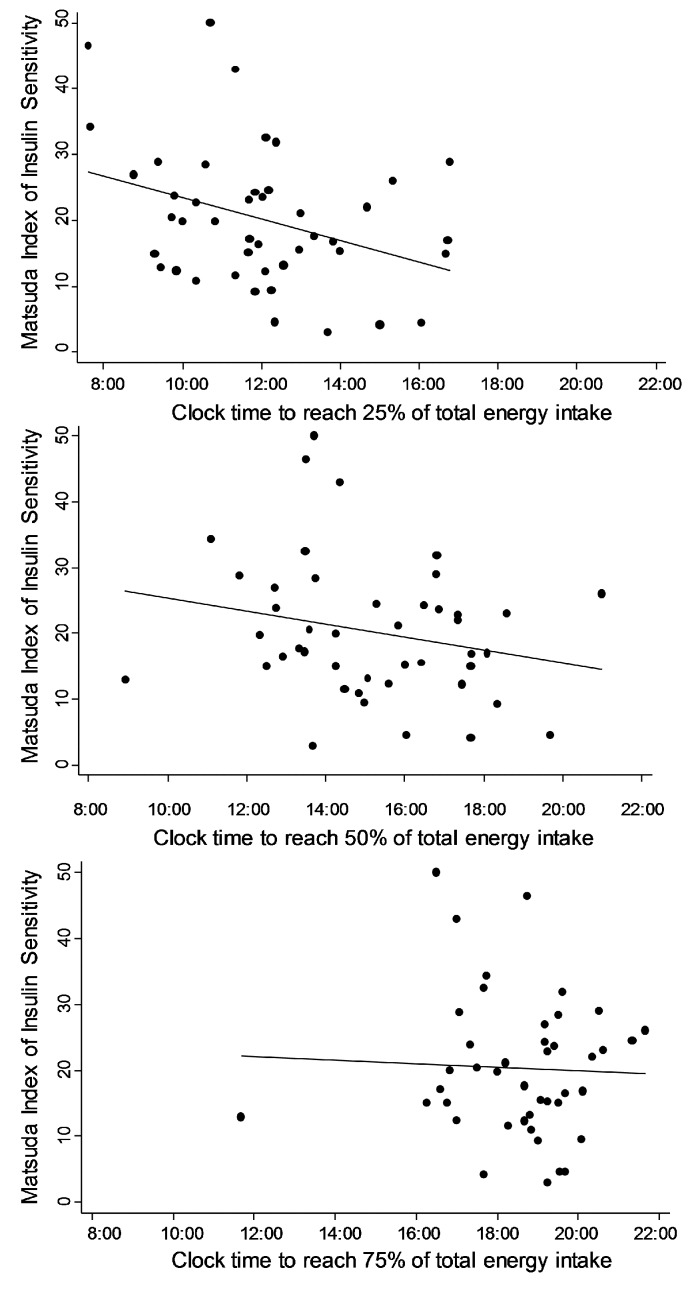
Associations between the Matsuda Index of insulin sensitivity and the clock time 25% of energy intake (**top panel**), 50% of energy intake (**middle panel**) and 75% of energy intake (**bottom panel**) was consumed. The unadjusted regression line is plotted. The r values represent unadjusted Pearson correlations and associated *p* values.

The clock time at which 25%, 50% and 75% of total energy was consumed demonstrated similar associations with insulin sensitivity (Table 3). Figure 2 illustrates these unadjusted associations using Matsuda Index of insulin sensitivity. The clock time at which 25% of daily energy intake was consumed was most strongly associated with insulin sensitivity. Every hour later that 25% was reached was associated with 1.4 units lower Matsuda Index and 0.004 units lower QUICKI in fully adjusted models. Note that using the log-transformed version of the time at which 75% of total energy consumed had similar results. Using Matsuda Index at 300 min (*n* = 40) produced similar results (see Supplemental Table S1). 

Among the macronutrients, the clock times at which 25% and 50% of the total daily carbohydrates were consumed were negatively associated with insulin sensitivity based on the Matsuda Index (B = −1.7, 95%CI −3.0, −0.3, *p* = 0.02 for 25% and B = −1.9, 95%CI −3.3, −0.6, *p* = 0.007 for 50% in the fully adjusted models) and QUICKI (B = −0.004, 95%CI −0.008, −0.001, *p* = 0.010 for 25% and B = −0.005, 95%CI −0.008, −0.001, *p* = 0.009 for 50% in the fully adjusted models). The time at which 75% of total protein intake was reached was inversely associated with Matsuda Index (B = −2.2, 95%CI −3.8, −0.5, *p* = 0.01) and this association was similar when using the log transformed variable (B = −2.2, 95%CI −3.8, −0.5, *p* = 0.01).

When we restricted our sample to those whose habitual average wake time was before the start of the OGTT (*n* = 39), the results of the unadjusted analyses were similar with respect to both effect size (regression coefficients) and statistical significance (see Supplemental Table S2). In the fully adjusted models, the effect sizes were similar, but some associations lost statistical significance (*p* > 0.05). The proportion of carbohydrates consumed in the morning remained significantly associated with both Matsuda Index (B = 0.27, 95%CI 0.02, 0.51, *p* = 0.03) and QUICKI (B = 0.0006, 95%CI 0.00002, 0.001, *p* = 0.04) in the fully adjusted models. The time at which 25% and 50% of total carbohydrate intake was reached remained significantly associated with insulin sensitivity based on QUICKI (B = −0.004, 95%CI −0.008, −0.0002, *p* = 0.04 for 25% and B = −0.005, 95%CI −0.009, −0.0005, *p* = 0.03 for 50%) but not Matsuda Index. The time at which 75% of total protein consumption was reached also remained significantly associated with Matsuda Index (B = −2.0, 95%CI −3.7, −0.3, *p* = 0.03).

## 4. Discussion

In the present study, we examined timing of meals in two different ways: proportion consumed in the morning and evening, which were defined by the participant, and the clock time at which specific proportions of total intake were reached. This latter method is a new metric that does not rely on subjective identification of a “breakfast” versus “lunch” to identify temporal caloric distribution using actual clock times. Furthermore, this metric will allow comparisons with other clock timing measures in future research. Both measures showed that energy intake, particularly carbohydrates, earlier in the day was associated with higher insulin sensitivity, even after controlling for potential confounders such as age, race, sex, BMI, average 24-h energy intake and sleep timing. 

These findings are consistent with literature reporting an association between breakfast skipping and increased insulin resistance [24,25] and type 2 diabetes [26,27]. For example, a small randomized crossover study of eight young men observed an increase in 24-h glucose levels without changes in energy expenditure following breakfast skipping compared to breakfast consumption [28]. Not all studies, however, found that adults who skipped breakfast were at an increased risk for diabetes. One limitation in this literature is the absence of a standard definition of breakfast. The definition of breakfast ranged from the first meal of the day, a meal consumed within 2 h of waking or energy intake anywhere between 5 am to 10 am regardless of when they woke [29,30]. In other studies, questionnaires that assessed breakfast consumption frequency was used to classify a participant as breakfast skipper (consumes breakfast three or less days/week) or not (consumes breakfast 5–7 days/week) [31,32]. Collectively these discrepancies in the definition of breakfast could result in differences in the degree of association between diabetes and breakfast skipping. To minimize the discrepancies in the definition of the morning energy intake, we developed a measure that identified the times energy intake occurred, specifically the clock time at which one consumed 25%, 50% or 75% of daily energy intake, and found that the 25% time point was most strongly associated with insulin sensitivity. This is an unbiased measure of temporal and quantitative energy intake distribution because it is independent of frequency of eating events and subjectivity of participants classifying an eating event as specific meal (e.g., “breakfast” or “snack”). 

Timing of eating has emerged as a dietary behavior with pronounced effects on metabolic health. A weight loss study in Spain observed that those who ate their primary meal before 15:00 (the median time) lost an average of approximately 2.2 kg more over 20 weeks than those who ate it later, despite no differences in reported food intake or physical activity [33]. A recent study has also shown that subjects who had higher percentage of body fat consumed calories later in their biological day (based on endogenous circadian phase) [14]. Several experimental studies that manipulated timing of food intake have observed significant changes in insulin sensitivity or dynamics. In a small randomized crossover study of 6 individuals, consuming 60% of daily calories in the evening lead to lower insulin sensitivity than when they consumed 60% calories in the morning [34]. Another study of 74 women with the metabolic syndrome involved 12 weeks of a caloric restricted diet where 50% of their daily calories were consumed either in the morning or in the evening [35]. At the end of 12 weeks, the morning group lost more weight and had greater improvements in insulin sensitivity than the evening group. An experimental study of meal skipping found that skipping breakfast, but not dinner, was associated with changes in insulin concentrations and fat oxidation suggestive of metabolic inflexibility [36]. Finally, one study randomized type 2 diabetes patients to either high-energy breakfast and reduced-energy dinner or the converse for 7 days and found that the high-energy breakfast was associated with lower postprandial glucose levels [37]. Together, these studies suggest a distribution towards more calories earlier in the day may have beneficial effects on metabolic function.

The effect of temporal distribution of energy intake on metabolism and insulin sensitivity may be explained by the circadian system, which has been linked with energy regulation and metabolism. For example, glucose tolerance and insulin sensitivity exhibit clear diurnal variations where higher glucose tolerance and insulin sensitivity occur during the early part of the biological day and then gradually declines to reach its nadir during the biological night [23]. In addition, feeding is a time cue that helps to synchronize peripheral clocks in tissues and therefore feeding at an inappropriate time could lead to misalignment between the central and peripheral clocks, which may have deleterious effects. The negative effect of eating at the wrong time of day is clearly illustrated in an animal study of mice that allowed food access either in the day (incorrect feeding time) or night (correct feeding time). Mice who ate only at the incorrect time gained substantially more weight than mice who ate at the correct time despite similar energy intake and physical activity [38]. Studies in rodent models have also demonstrated that the timing of food consumption as well as the amount consumed at specific meals is under control of the circadian system [39,40]. Notably, when food availability is limited to only four opportunities in a 12-h period, the amount consumed in the last “meal” is the lowest, even if the animal is experiencing weight loss. These findings are consistent with our finding that earlier energy consumption may be preferable and together these studies further implicate the important role of the circadian system in meal timing and distribution.

Our study included careful screening and exclusion for existing diabetes and sleep disorders, assessment and adjustment for habitual sleep timing, as well as the assessment of insulin sensitivity using both a glucose tolerance test and fasting measures to confirm associations. We also created a dietary timing variable that was independent of subjective definition of meals in the food records. There are some limitations to acknowledge, however. The primary limitation is the cross-sectional design because causality cannot be determined. Second, food records may lead to underreporting of caloric intake, as with other dietary assessment methods in free-living humans. However, the degree to which underreporting affects the assessment of the timing of energy intake is unknown. Unfortunately, there are no objective measures of food intake and timing that do not rely on participant reporting or input. Another potential limitation is that the OGTT was conducted in the morning for all participants. It is possible that insulin sensitivity may be higher later in the day in people who habitually consume more food, particularly carbohydrates, later in the day. Future research could be designed to test this possibility. Finally, these results may not be generalizable to people with diabetes or other patient populations.

## 5. Conclusions

The 2015 Dietary Guidelines for Americans (DGA) committee concluded that an overall healthy eating pattern is necessary to maintain good health and reduce chronic disease risk [41]. The components of dietary patterns can have a potentially cumulative effect on health, and it is therefore necessary to characterize a healthy dietary pattern, which would certainly include the quantity and quality of the diet. More recent evidence, including our findings, support inclusion of the timing of meals. Our study suggests that consuming 25% of your total energy intake earlier in the day is associated with higher insulin sensitivity, irrespective of the total amount of calories consumed—even in people without diabetes. A similar pattern was also established for carbohydrates consumed. Whether those who consume more calories or carbohydrates earlier in the day will have a reduced risk of diabetes remains to be seen, but these findings lend additional support to exploring dietary interventions that focus on timing as well as content.

## Figures and Tables

**Table 1:** Clinical characteristics, sleep parameters and glucose metabolism parameters of study participants (*n* = 44).

	Mean ± SD or *n* (%)
Age (years)	29.8 ± 7.6
Sex	
Male	23 (52%)
Female	21 (48%)
Race	
African American	17 (39%)
Non-Hispanic White	27 (61%)
BMI (kg/m^2^)	27.1 ± 5.1
Glucose metabolism parameters	
Fasting glucose (mmol/L)	4.8 ± 0.4
Fasting insulin (pmol/L)	50.7 ± 56.9
Matsuda Index—180 min	20.3 ± 10.5
Matsuda Index—300 min (*n* = 43)	25.8 ± 13.9
QUICKI	0.2 ± 0.03
Sleep timing	
Sleep start	00:14 ± 1:24
Sleep end	7:34 ± 1:24
Sleep midpoint	03:40 ± 0:21

**Table 2:** Dietary characteristics of study participants (*n* = 44).

	Mean ± SD
Dietary Proportion Measures	
Total calories per day (Kcal)	2411 ± 744
Proportion of total calories	
In morning meals (% Kcal)	23.1 ± 12.3
In evening meals (% Kcal)	40.5 ± 12.9
Proportion of carbohydrates	
In morning meals (% Kcal)	26.2 ± 13.7
In evening meals (% Kcal)	37.1 ± 11.8
Proportion of protein	
In morning meals (% Kcal)	20.8 ± 11.5
In evening meals (% Kcal)	42.5 ± 15.1
Proportion of fat	
In morning meals (% Kcal)	22.7 ± 14.6
In evening meals (% Kcal)	39.5 ± 16.2
Dietary Timing Measures	
Timing of total calories	
Time reached 25%	11:59 ± 2:17
Time reached 50%	15:12 ± 2:26
Time reached 75%	18:35 ± 1:42
Timing of carbohydrates	
Time reached 25%	11:45 ± 2:15
Time reached 50%	14:40 ± 2:05
Time reached 75%	18:17 ± 1:55
Timing of protein	
Time reached 25%	12:25 ± 2:03
Time reached 50%	15:21 ± 2:20
Time reached 75%	18:23 ± 1:47
Timing of fat	
Time reached 25%	12:29 ± 2:21
Time reached 50%	15:17 ± 2:31
Time reached 75%	17:42 ± 2:21

**Table 3:** Associations between insulin sensitivity and dietary proportion variables using linear regression models (*n* = 44).

Dependent Variable	Independent Variable	Unadjusted Model		Adjusted Model *	
		B-Coefficient (95% CI)	*p*-Value	B-Coefficient (95% CI)	*p*-Value
Proportions					
Matsuda Index	Proportion of morning calories (per 10%)	3.3 (0.8 to 5.8)	0.011	2.8 (0.3 to 5.2)	0.03
	Proportion of evening calories (per 10%)	−1.7 (−4.2 to 0.8)	0.17	−1.9 (−4.4 to 0.6)	0.13
QUICKI	Proportion of morning calories (per 10%)	0.008 (0.001 to 0.013)	0.018	0.006 (−0.0002 to 0.012)	0.057
	Proportion of evening calories (per 10%)	−0.003 (−0.009 to 0.004)	0.39	−.003 (−0.009 to 0.003)	0.28
Timing					
Matsuda Index	25% total calorie intake (per hour)	−1.6 (−3.0 to −0.3)	0.02	−1.4 (−2.8 to −0.1)	0.04
	50% total calorie intake (per hour)	−1.0 (−2.3 to 0.3)	0.14	−1.0 (−2.3 to 0.2)	0.10
	75% total calorie intake (per hour)	−0.3 (−2.2 to 1.7)	0.77	−0.9 (−2.9 to 1.0)	0.34
QUICKI	25% total calorie intake (per hour)	−0.004 (−0.008 to −0.001)	0.01	−0.004 (−0.007 to −0.001)	0.02
	50% total calorie intake (per hour)	−0.002 (−0.006 to 0.001)	0.14	−0.002 (−0.006 to 0.001)	0.11
	75% total calorie intake (per hour)	−0.001 (−0.006 to 0.004)	0.70	−0.002 (−0.007, 0.003)	0.41

* The models were adjusted for age, sex, BMI, race, total daily energy intake and sleep timing.

## Supplementary Materials

The following are available online at https://www.mdpi.com/2072-6643/12/2/503/s1. Table S1: Associations between insulin sensitivity estimated using the Matsuda Index calculated with 300 minutes and dietary proportion variables using linear regression models (*n* = 40). Table S2: Associations between insulin sensitivity and dietary proportion variables using linear regression models in those whose habitual wake time is earlier than start of OGTT (*n* = 39).
