# Supplementary material for: Association between Timing of Energy Intake and Insulin Sensitivity: A Cross-Sectional Study
Source: Nutrients. 2020 Feb 16;12(2):503. doi: 10.3390/nu12020503 (PMC7071301; doi:10.3390/nu12020503)
Supplement: Supplementary file 1 [file nutrients-12-00503-s001.pdf]

Supplemental Material for Rangaraj *et al*, Association between timing of energy intake and insulin sensitivity: a cross-sectional study

Supplemental Table S1: Associations between insulin sensitivity estimated using the Matsuda Index calculated with 300 minutes and dietary proportion variables using linear regression models (n=40).

**Dependent Variable**	**Independent Variable**	**Unadjusted Model**		**Adjusted Model** *	
		B-coefficient (95% CI)	P-value	B-coefficient (95% CI)	P-value
Proportions					
Matsuda Index – 300 minutes	**Proportion of morning calories (per 10%)**	**4.1 (0.7 to 7.5)**	**0.020**	**3.7 (0.3 to 7.1)**	**0.03**
	Proportion of evening calories (per 10%)	-2.4 (-5.8 to 0.9)	0.15	-3.1 (-6.5 to 0.3)	0.07
Timing					
Matsuda Index – 300 minutes	**25% total calorie intake (per hour)**	**-2.2 (-4.0 to -0.3)**	**0.023**	**-2.2 (-4.1 to -0.4)**	**0.02**
	50% total calorie intake (per hour)	-1.5 (-3.3 to 0.4)	0.12	-1.7 (-3.4 to 0.1)	0.07
	75% total calorie intake (per hour)	-0.4 (-3.0 to 2.3)	0.79	-1.3 (-4.0 to 1.4)	0.33

* The models were adjusted for age, sex, BMI, race, total daily energy intake and sleep timing.

Supplemental Table S2: Associations between insulin sensitivity and dietary proportion variables using linear regression models in those whose habitual wake time is earlier than start of OGTT (n=39).

**Dependent Variable**	**Independent Variable**	**Unadjusted Model**		**Adjusted Model** *	
		B-coefficient (95% CI)	P-value	B-coefficient (95% CI)	P-value
Proportions					
Matsuda Index	**Proportion of morning calories (per 10%)**	**3.3 (0.5 to 6.2)**	**0.021**	2.1 (-0.8 to 5.0)	0.15
	Proportion of evening calories (per 10%)	-1.9 (-4.8 to 1.1)	0.21	-1.2 (-4.1 to 1.7)	0.40
QUICKI	**Proportion of morning calories (per 10%)**	**0.008 (.001 to 0.015)**	**0.02**	0.005 (-0.002 to 0.012)	0.15
	Proportion of evening calories (per 10%)	-0.003 (-0.01 to 0.005)	0.46	-.002 (-0.009 to 0.005)	0.61
Timing					
Matsuda Index	**25% total calorie intake (per hour)**	**-1.9 (-3.5 to -0.3)**	**0.025**	-1.0 (-2.7 to 0.7)	0.24
	50% total calorie intake (per hour)	-0.9 (-2.4 to 0.6)	0.23	-0.7 (-2.1 to 0.7)	0.35
	75% total calorie intake (per hour)	-0.2 (-2.3 to 1.9)	0.85	-0.6 (-2.6 to 1.5)	0.56
QUICKI	**25% total calorie intake (per hour)**	**-0.005 (-0.009 to -0.001)**	**0.01**	-0.004 (-0.008 to 0.0004)	0.07
	50% total calorie intake (per hour)	-0.002 (-0.006 to 0.001)	0.18	-0.002 (-0.005 to 0.002)	0.28
	75% total calorie intake (per hour)	-0.001 (-.006 to .004)	0.74	-0.001 (-0.007, 0.004)	0.60

* The models were adjusted for age, sex, BMI, race, total daily energy intake and sleep timing.
